# Novel Mutations Mapping to the Fourth Sodium Channel Domain of Nav1.7 Result in Variable Clinical Manifestations of Primary Erythromelalgia

**DOI:** 10.1007/s12017-012-8216-8

**Published:** 2013-01-06

**Authors:** Roman Cregg, Bisola Laguda, Robert Werdehausen, James J. Cox, John E. Linley, Juan D. Ramirez, Istvan Bodi, Michael Markiewicz, Kevin J. Howell, Ya-Chun Chen, Karen Agnew, Henry Houlden, Michael P. Lunn, David L. H. Bennett, John N. Wood, Maria Kinali

**Affiliations:** 1 Molecular Nociception Group, Wolfson Institute for Biomedical Research, UCL, Gower Street, London, WC1E 6BT UK; 2 UCL Centre for Anaesthesia, Critical Care and Pain Medicine, London, UK; 3 Department of Paediatric Dermatology, Chelsea and Westminster Hospital, London, UK; 4 Department of Anesthesiology, Heinrich-Heine-University, Düsseldorf, Germany; 5 Neurorestoration Group, CARD, King’s College London, Guy’s Campus, London, UK; 6 Department of Clinical Neuropathology, King’s College Hospital, London, UK; 7 Department of Paediatrics, Chelsea and Westminster Hospital, London, UK; 8 Centre for Rheumatology and Connective Tissue Disease, UCL Division of Medicine, Royal Free Campus, London, UK; 9 Division of Cell and Molecular Biology, Faculty of Natural Sciences, Imperial College London, London, UK; 10 MRC Centre for Neuromuscular Diseases, UCL Institute of Neurology and The National Hospital for Neurology and Neurosurgery, Queen Square, London, UK; 11 Oxford Neuroscience, University of Oxford, Oxford, UK; 12 Department of Paediatric Neurology, Chelsea and Westminster Hospital, London, UK; 13 The Nuffield Department of Clinical Neurosciences, University of Oxford, John Radcliffe Hospital, Oxford, UK

**Keywords:** Erythromelalgia, Neuropathic pain, Voltage-gated sodium channels, Gain-of-function mutations, Nav1.7

## Abstract

We identified and clinically investigated two patients with primary erythromelalgia mutations (PEM), which are the first reported to map to the fourth domain of Nav1.7 (*D*IV). The identified mutations (A1746G and W1538R) were cloned and transfected to cell cultures followed by electrophysiological analysis in whole-cell configuration. The investigated patients presented with PEM, while age of onset was very different (3 vs. 61 years of age). Electrophysiological characterization revealed that the early onset A1746G mutation leads to a marked hyperpolarizing shift in voltage dependence of steady-state activation, larger window currents, faster activation kinetics (time-to-peak current) and recovery from steady-state inactivation compared to wild-type Nav1.7, indicating a pronounced gain-of-function. Furthermore, we found a hyperpolarizing shift in voltage dependence of slow inactivation, which is another feature commonly found in Nav1.7 mutations associated with PEM. In silico neuron simulation revealed reduced firing thresholds and increased repetitive firing, both indicating hyperexcitability. The late-onset W1538R mutation also revealed gain-of-function properties, although to a lesser extent. Our findings demonstrate that mutations encoding for DIV of Nav1.7 can not only be linked to congenital insensitivity to pain or paroxysmal extreme pain disorder but can also be causative of PEM, if voltage dependency of channel activation is affected. This supports the view that the degree of biophysical property changes caused by a mutation may have an impact on age of clinical manifestation of PEM. In summary, these findings extent the genotype–phenotype correlation profile for *SCN9A* and highlight a new region of Nav1.7 that is implicated in PEM.

**Electronic supplementary material:**

The online version of this article (doi:10.1007/s12017-012-8216-8) contains supplementary material, which is available to authorized users.

## Introduction

Recent studies have confirmed a pivotal role for the Nav1.7 voltage-gated sodium channel in human familial gain-of-function (Yang et al. 2004; Fertleman et al. 2006) and loss-of-function pain syndromes (Cox et al. 2006). Nav1.7, encoded by SCN9A, is preferentially expressed in nociceptive dorsal root ganglia (DRG) and sympathetic ganglia (Sangameswaran et al. 1997; Toledo-Aral et al. 1997) and is thought to serve a threshold triggering function, enabling depolarizing stimuli to elicit action potential propagation (Cummins et al. 1998; Rush et al. 2007). By identifying patients with novel mutations in SCN9A and analyzing the biophysical properties of the mutant Nav1.7 channels, we aim to provide insights into how this important channel functions and contributes to neuronal action potential firing, potentially suggesting ways in which its function could be normalized in affected individuals.

Recessive loss-of-function mutations in SCN9A result in congenital insensitivity to pain (CIP), whereas gain-of-function, dominant mutations lead to sensory neuronal hyperexcitability and the development of painful phenotypes described as inherited or primary erythromelalgia (PEM or IEM) (Yang et al. 2004; Dib-Hajj et al. 2005) and paroxysmal extreme pain disorder (PEPD) (Fertleman et al. 2006; Choi et al. 2011). PEM is classically described as a peripheral, bilateral disorder associated with erythema and severe burning pain of affected extremities that is triggered by stress, exhaustion and warmth and often relieved by cooling of the affected sites (Segerdahl et al. 2012). Clinical onset has been reported previously as within 1 year after birth in some cases, while in the majority of cases, symptoms of PEM develop within the first decade of life (Fischer and Waxman 2010). With no medication for Nav1.7-specific sodium channel block available, options for pharmacologic treatment are limited to systemic application of non-specific sodium channel blocking agents, for example, lidocaine (Sheets et al. 2007) and mexiletine (Choi et al. 2009), and symptomatic approaches for neuropathic pain treatment including anticonvulsants, opioids and antidepressants (Hisama et al. 2006). Further elucidation of the connection between single-point mutations in SCN9A encoding for Nav1.7 gain-of-function mutations, impact on biophysical channel properties, disease onset and clinical symptoms may contribute to our understanding of this debilitating disease, and enable us to interpret results from genetic testing more rationally and to develop a causative treatment.

We report a detailed clinical characterization and biophysical analysis of two novel mutations in Nav1.7, which are associated with PEM. Interestingly, while the described mutations are the first reported PE mutations to map to the fourth domain of Nav1.7, they are associated with very different clinical characteristics with one patient having a documented onset of symptoms at 3 years of age and the other patient having an extremely late age of onset at 61 years of age. These clinical differences might be explained by the observed different biophysical properties of the two mutated Nav1.7 channels.

## Materials and Methods

### Standard Protocol Approvals, Registrations and Patient Consents

Informed consent was given by both investigated patients for genetic testing as well as for publication of results and clinical case histories. Patient 1 was recruited as part of the IRB-approved Pain in Neuropathy Study (National Research Ethics Service Ref. 10/H07056/35), which included quantitative sensory testing (QST) on a research basis.

### Quantitative Sensory Testing

QST following informed consent was performed on the dorsum of the foot and the hand according to the German DFNS protocol (Rolke et al. 2006). This protocol includes 13 parameters measuring temperature detection and pain thresholds, mechanical detection and pain thresholds, mechanical pain sensitivity, and signs of allodynia and wind-up ratio among others, allowing characterization of sensory profiles. The findings in the two evaluated patients were compared with a control Caucasian population by means of *Z* scores in which the *z* score represents the result of a raw score minus the mean of the population and this is further divided by the standard deviation of the population. *Z* scores above or below ±2 standard deviations represent hyper-/hypo-sensitivity or hyper-/hypo-algesia depending on the evaluated parameter.

### SCN9A Sequencing

Genomic DNA was isolated from blood by standard methods. All coding exons and flanking splice sites of SCN9A were bidirectionally sequenced as part of clinical diagnostic investigation (Dept. Gastroenterology, Radboud University, Nijmegen Medical Centre, The Netherlands) and compared to the human genome sequence (build 37) at http://genome.ucsc.edu. In patient 1, a heterozygous c.4612t>c variant (W1538R) was found, and in patient 2, a heterozygous c.5237C>G variant (A1746G) was found. Both variants are absent from the latest dbSNP database release (dbSNP 135). Nucleotide numbering reflects cDNA numbering with +1 corresponding to the A of the ATG translation initiation codon in the reference sequence (Ref Seq) nm_002977.3. An in silico prediction of the functional effects of the 2 non-synonymous variants was performed using PolyPhen-2 and SIFT (http://genetics.bwh.harvard.edu/pph2/ and http://sift.jcvi.org, respectively).

### Plasmid and Site-Directed Mutagenesis

A previously described full-length human SCN9A cDNA sequence cloned into a modified pcDNA3 expression vector containing downstream polio IRES and DsRED2 sequences (FLRED) was used (Cox et al. 2006). The patient mutations were introduced into FLRED using the QuikChange II XL Site-Directed Mutagenesis Kit (Stratagene) according to the manufacturer’s instructions. The coding sequence of both constructs was fully sequenced to verify the desired mutation and to ensure the lack of other introduced variations.

### Cell Culture and Transfection

Human embryonic kidney cells (HEK293A) were cultured in a humidified atmosphere containing 5 % carbon dioxide at 37 °C and were grown in Dulbecco’s modified Eagle’s medium (DMEM; Gibco, Life Technologies, Carlsbad, CA) supplemented with 10 % heat-inactivated fetal bovine serum. Unless stated otherwise, reagents were purchased from Sigma-Aldrich (St. Louis, MO).

Cells were transiently transfected with plasmid DNA for expression of Nav1.7 human wild-type α subunits (Ref Seq NM_002977), A1746G or W1538R-mutated α subunits (SCN9A-IRES-DsRed2 in pcDNA3 vector) combined with human wild-type β1 (Ref Seq NM_001037) and β2 (Ref Seq NM_001037) subunits (SCN1B-IRES-SCN2B-IRES-eGFP in a pIRES2-AcGFP1 backbone vector) as previously described (Cox et al. 2006). In brief, transient transfection was performed with cells seeded at 80–90 % confluency in 35-mm cell culture dishes using Lipofectamine 2000 (Invitrogen, Life Technologies) according to the manufacturer’s recommendations. After 6 h, transfection medium was replaced with fresh culture medium and the cells were re-seeded for electrophysiological recordings at 20–30 % confluency.

### Electrophysiological Recordings

Whole-cell membrane current recordings were performed 46–78 h after transfection. All recordings were made at room temperature. Micropipettes were pulled from borosilicate glass capillaries (GC150F-10; Harvard Apparatus, Kent, UK) using a Brown-Flaming P-97 horizontal micropipette puller (Sutter Instruments, Novato, CA, USA) and then fire polished on a microforge (MF-830 Narishige Group, Tokyo, Japan). Voltage errors were minimized with correction and prediction mode of series resistance compensation both set to 50 %. Extracellular solution contained (in mmol/L) 140 NaCl, 4 KCl, 2 CaCl_2_, 1 MgCl_2_, 10 HEPES, adjusted to pH 7.4 with NaOH, osmolarity 320–325 mOsm/L with glucose. Pipettes were filled with an intracellular solution containing (in mmol/L) 140 CsCl, 5 NaCl, 5 EGTA, 2 MgCl_2_, 10 HEPES adjusted to pH 7.3 with CsOH, osmolarity 305–310 mOsm/L with glucose. Once filled with the appropriate intracellular solution, recording electrodes had a resistance between 2.0 and 3.2 MΩ. A silver chloride-coated silver wire served as a reference electrode with one end connected to the ground input of the amplifier and the tip placed directly into the bath solution. Cells having a leak current after establishing whole-cell configuration of more than 10 % of the peak sodium current were discarded and those which had developed leak of this magnitude during the experiment were not used in the final analysis. The liquid junction potential between the bath and the pipette solutions was not corrected. Whole-cell membrane currents were filtered at 5 kHz and sampled at 20 kHz using an Axopatch 200B patch clamp amplifier (Molecular Devices, Foster City, CA) and Digidata 1200B A/D converter (Molecular Devices, Foster City, CA). Data were acquired on a Windows-based PC using Clampex software (Molecular Devices, Foster City, CA) and analyzed by pCLAMP (Clampfit) 9.2 software (Molecular Devices, Foster City, CA).

### Voltage-Clamp Protocols

To characterize the voltage dependency of steady-state channel activation, currents were evoked by voltage increments of 5 mV from −80 to +70 mV from a holding potential of −120 mV (Cummins et al. 2009). Persistent currents were quantified at 8 ms after onset of the depolarizing voltage step (Fertleman et al. 2006; Dib-Hajj et al. 2008; Jarecki et al. 2008). Conductance (*G*) values were calculated from measured peak inward currents (*I*) and observed reversal potential for sodium ions (*E*_rev Na_) using the equation *G* = *I*/(*V*_m_ − *E*_rev Na_).

Reversal potential was measured by extrapolating the linear portion of the *I*/*V* relationship between +15 and +60 mV. Resulting values for conductance were normalized to the peak inward current and fitted using the Boltzmann equation. Steady-state inactivation of wild-type Nav1.7 channels, W1538R or A1746G mutations was assessed by holding cells at potentials incrementing from −110 mV to 0 mV for 500 ms, followed by a step to −10 mV for 50 ms. Voltage dependence of slow inactivation was investigated using a 20-ms pulse to 0 mV after a 10-s pre-pulse to potentials from −100 to 0 mV followed by a 100-ms pulse to −120 mV to remove fast inactivation. Resulting I–V curves were then fitted by using the Boltzmann equation. With a preconditioning pulse of 10 s, no complete slow inactivation was achieved; therefore, resulting values may not compare to previously published data for slow inactivation in WT Nav1.7. However, a time window of 10 s is considered to be relevant for inducing slow inactivation (Estacion et al. 2010), and therefore, differences seen in mutated Nav1.7 channels compared to WT are representative of enhanced slow inactivation. To investigate the time to recovery from fast inactivation, first a 20-ms pulse to −10 mV was applied followed by a recovery phase between 5 and 100 ms and a second pulse to −10 mV (Cummins et al. 2009). Recorded peak currents were then normalized and fitted using a single exponential equation (one-phase association).

### Statistical Analysis

All data are expressed as mean ± SEM. Differences in means between wild-type channels and mutations were tested by two-tailed Student’s *t* test or one-way ANOVA with the Bonferroni post-test where appropriate. *P* < 0.05 was considered significant. Calculations were made using the Graph Pad Prism Software version 5.0 (GraphPad Software Inc., La Jolla, CA).

## Results

### Clinical Presentation: Patient 1

The first case is a 68-year-old woman who first developed bilateral foot pain at the age of 61. The pain was mild at onset, however, progressively worsened in severity and she subsequently noted erythema of the feet. She currently suffers from constant pain affecting particularly the soles and heels of her feet and extending to the level of her ankles. She typically rates this pain as 8 out of 10 on a numerical rating scale; however, this can increase to 10 out of 10 during exacerbations. The pain is described as burning, and at times, she feels that her feet are about to burst. She is never pain free. Exacerbations are triggered by heat, prolonged standing and wearing enclosed shoes. These exacerbations last a number of hours, are associated with increased foot erythema and can be relieved by cooling. She scored 7 out of 10 on the DN4 screening questionnaire for neuropathic pain (suggesting pain symptomatology consistent with a neuropathic etiology) (Bouhassira et al. 2005). She is significantly disabled by her pain and now requires a wheelchair. She notes occasional paresthesia and numbness of the feet. There is no history of foot ulceration or autonomic symptoms. There may be a relevant family history in that her mother who is deceased complained of foot pain, which limited her ability to mobilize.

### Examination and Investigations: Patient 1

**Fig. 1:**
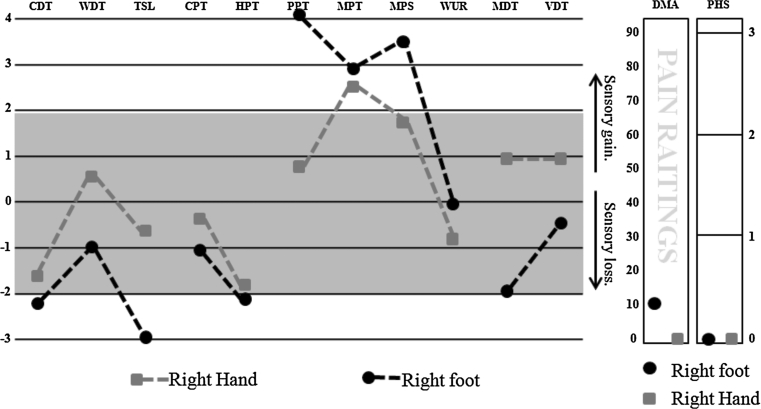
Results of quantitative sensory testing in patient 1. Comparisons of evaluated sites are made against a Caucasian control population for each one of the evaluated sites, and tested sites differences are remarked below. Gain of sensory function is presented as a *z* score >2, and loss of sensory function as <2. CDT cold detection thresholds. *WDT* warm detection thresholds, *TSL* thermal sensory limen, *CPT* cold pain thresholds, *HPT* heat pain thresholds, *PPT* pressure pain thresholds, *MPT* mechanical pain thresholds, *MPS* mechanical pain sensitivity, *WUR* wind-up ratio, *MDT* mechanical detection thresholds, *VDT* vibration detection thresholds, *DMA* dynamic mechanical allodynia, *PHS* paradoxical heat sensations. Test sites: right hand and right foot. In the right foot, which was the symptomatic region, there was evidence of both gain and loss of sensory function. Specifically, we noted cold and thermal sensory limen hypoesthesia and heat pain hypoalgesia. There was evidence of mechanical hypersensitivity including pressure and mechanical pain hyperalgesia as well as mechanical pain hypersensitivity and dynamic mechanical allodynia

On clinical examination, erythema of the forefoot was noted. Her gait was hesitant due to foot pain; otherwise, motor function was normal. Deep tendon reflexes, light touch, vibration sense and proprioception were normal. There was a subjective reduction in pinprick sensibility to the ankles. Nerve conduction studies of the upper and lower limbs were normal, and EMG of the peroneus tertius muscle was normal. Quantitative sensory testing of her right hand (which was asymptomatic) and right foot (symptomatic) was performed as per DFNS protocol (Rolke et al. 2006). It should be noted that in the hand, findings were normal other than hyperalgesia to high threshold mechanical stimuli (Fig. 1). In the feet, which were symptomatic, there was a mixture of both loss and gain of sensory function. There was reduced sensitivity to cooling and thermal sensory limen but enhanced sensitivity to mechanical stimuli including pressure pain, mechanical pain and dynamic mechanical allodynia (Fig. 1).

On testing autonomic function, there was no orthostatic hypotension. The pressor responses to mental arithmetic tasks and cutaneous cold stimuli were present, although there was a minimal response to isometric exercise. Respiratory sinus arrhythmia was present, while heart rate response to hyperventilation was minimal. A well-performed Valsalva maneuver yielded normal blood pressure profile. Overall, there was no evidence for cardiovascular autonomic dysfunction. The sympathetic skin response in the feet was normal (422 μV amplitude, 2,315 ms latency). Blood tests including full blood count, plasma protein electrophoresis, erythrocyte sedimentation rate, C-reactive protein, renal profile, liver profile, thyroid function tests, glucose, vitamin B12 levels, hepatitis B and C serology, anti-nuclear antibodies and cryoglobulins did not show any significant abnormalities. Skin biopsy of the distal leg was performed as described previously (Ramirez et al. 2012) in order to investigate the possibility of a small fiber neuropathy. This demonstrated a normal intra-epidermal nerve fiber density of 11.93 fibers/mm as assessed by immunostaining for protein gene product 9.5 according to the guidelines of the European Federation of Neurological Societies (Lauria et al. 2010).

### Treatment: Patient 1

She received partial benefit from treatment with gabapentin (900 mg; three times a day) and in addition used tramadol (50–150 mg) as rescue medication. A large number of alternative medications have not provided pain relief including carbamazepine, amitriptyline, duloxetine as well as 8 % capsaicin and 5 % lidocaine transdermal systems applied to the feet.

### Clinical Presentation: Patient 2

A White Caucasian boy aged 7 presented with a 4-year history of painful feet, which progressively worsened. It was described as episodic, burning pain affecting the soles and less so extending to the dorsal aspect of both feet. He rated the pain as 10 out of 10 on a numerical scale, typically lasting 15 min but on other occasions up to 24 h. The pain was particularly severe following exercise such as playing football or prolonged standing. It was also triggered by ambient hot temperature and wearing enclosed shoes. During exacerbations, he was unable to stand on his feet. He required cold air to be blown over his feet or had his feet hanging out of the car window, stuck on the window screen or submerged in cold water. He could walk up to 300 m. He wore open-toe shoes or more often ambulated barefoot. More recently, his hands have become affected and have complained of diffuse pains on his shoulders, knees and arms albeit infrequently (not more than once a month). The pain has never resulted in episodic stiffness, paralysis or dystonic posturing. He has never had any myoglobinuria, cramps, foot ulceration or dysautonomic symptoms. He did not suffer from allodynia or hyperalgesia. He had difficulty in concentrating at school.

The patient is the third child of non-consanguineous parents. In his family history, his father is known to suffer from non-organic, overwhelming aggression and high tolerance to pain with his paternal grandmother was diagnosed with a late-onset neuropathy. There were no neonatal concerns or feeding difficulties. His early development was normal. He had a personal history of early infantile seizures, one associated with fever.

### Examination and Investigations: Patient 2

Examination revealed a well-grown boy with normal physical examination. He ambulated barefoot with no limitation to his motor function. Tandem gait was equivocal (slight in coordination). It was noted that he had initially subtle erythema of his feet. Deep tendon reflexes, light touch, proprioception, vibration and two-point discrimination were all normal. He had no tremor, muscle atrophy or hypertrophy or orthostatic hypotension. Nerve conduction velocities studies showed no evidence of large fiber peripheral neuropathy, and EMG studies of the tibial anterior muscle (deep fibular nerve) were normal. Thermography revealed a rapid hyperemic response to cold challenge after cooling (Supplementary material 1). There was no cardiovascular autonomic dysfunction, and heart echocardiogram and EKG were both normal. He has had both genetic and psychological review, the latter in view of his debilitating symptoms and lack of concentration. Further investigations including baseline bloods, autoimmune screen, plasma and urine amino acids, troponin I, creatine kinase, carnitine profile, lactate, ammonia, vitamin E, cholesterol, triglyceride, B12 and red cell folate test, urine organic acids, EEG, brain CT, MRI of the brain and whole spine were normal.

### Treatment: Patient 2

Initially, he was treated with topical 0.025 % capsaicin cream on both feet with minimal improvement. He was subsequently started on mexiletine on a dose of 50 mg once daily, which was gradually increased to 100 mg three times daily (10 mg/kg/day). Treatment with mexiletine was started in association with 5 % lidocaine patches applied to the feet at night time over 12-h periods. His symptoms have gradually improved such that he now is able to walk to school and take part in sporting activities. His concentration has improved, and he is able to sleep at night without disturbance. He no longer submerges his feet in ice-cold water. The quality of life of the patient and his family has improved substantially.

### SCN9A Mutations Identified in Patients 1 and 2

Given the phenotype of erythromelalgia seen in the patients, albeit extremely late onset in patient 1, we decided to sequence SCN9A as this was the best candidate gene for the disorder in both patients. In patient 2, DNA sequencing of genomic DNA revealed a heterozygous missense variant, c.5237C>G, which is predicted to change amino acid 1746 of Nav1.7 from an alanine to a glycine (A1746G). This variant occurred de novo as both parents were shown to not carry this variant when sequenced. In silico analysis of this non-synonymous variant using PolyPhen-2 and SIFT tools gave scores of 0.999 and 0, respectively, indicating that both algorithms predict the mutation to be probably damaging. The mutation was absent from the most recent dbSNP database and not previously reported as a disease-causing mutation.

In patient 1, DNA sequencing identified a heterozygous missense variant, c.4612T>C, which is predicted to change amino acid 1538 from a tryptophan to an arginine (W1538R). The parents of the patient were both deceased. The variant was absent from the most recent dbSNP database. In silico analyses using PolyPhen-2 and SIFT gave scores of 0.001 and 0.85, respectively, indicating that the mutation is benign. However, the same mutation was previously reported to cause a late-onset chronic non-paroxysmal neuropathic pain phenotype (Dabby et al. 2011), although no biophysical analysis was performed for the mutation (note that in the publication of Dabby et al., the mutation is annotated as W1550R due to a different cDNA sequence used as the reference sequence). Patient 1 was also a heterozygous carrier of 4 additional SCN9A variants, which are annotated as SNPs in dbSNP (rs71428908, rs12478318, rs4369876 and rs3750904). Of note, a patient with small fiber neuropathy has previously been reported who is a compound heterozygote for rs12478318 and rs4369876 (Faber et al. 2012).

**Fig. 2:**
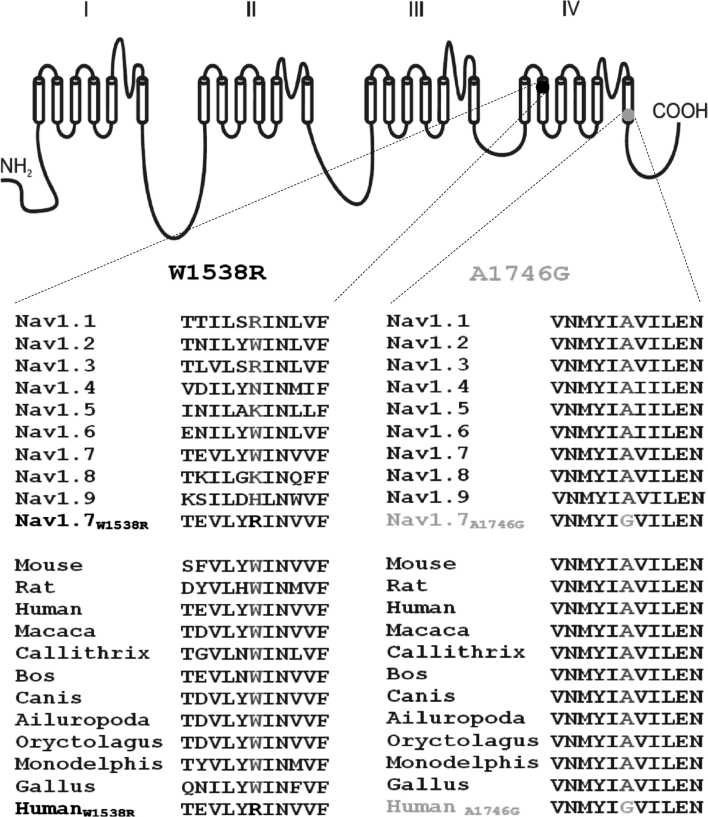
Amino acid residue alignment demonstrating magnitude of inter-isoform and inter-species conservation of the affected regions. *Blue color* residues identify the mutant W1538R (patient 1) and *red color* indicates A1746G mutation (patient 2) mapping to S2 DIV and S6 DIV of human Nav1.7α subunit, respectively. *Green color* indicates corresponding non-mutated variant

The A1746G and W1538R mutations both map to the fourth sodium channel domain of Nav1.7 (Fig. 2), where mutation A1746G maps to the sixth transmembrane segment, while mutation W1538R maps to the second transmembrane segment. Sequence alignment of voltage-gated sodium channels (Nav) indicated that the alanine at position 1746 is unilaterally conserved in every member of the Nav family in humans. Additionally, it is also highly evolutionarily conserved within each Nav1.7 channel across species. The late-onset mutation, W1538R, also shows high conservation across species but does not show such high conservation within the human Nav family with the mutant amino acid (arginine) found in both Nav1.1 and Nav1.3. We were therefore very keen to test the biophysical properties of these channels to determine whether each mutation was likely to be disease causing.

### Mutation of A1746G and W1538R Leads to a Hyperpolarizing Shift in the Current–Voltage (I–V) Relationship

The functional effect of A1746G and W1538R mutations on Nav1.7 function was investigated using the whole cell patch clamp technique on HEK cells transiently transfected with Nav1.7α (WT or mutant) + Navβ1 + Navβ2.

Peak currents and whole cell capacitance in HEK293 cells transfected with Nav1.7α subunit containing the mutation A1746G (*n* = 18) or W1538R (*n* = 14) in combination with Navβ1 and Navβ2 subunits did not significantly differ from cells with wild-type Nav1.7 (*n* = 20) (Table 1).

**Table 1:** Cell and recording properties

	Peak current (nA)	WCC (pF)	Series resistance (MΩ)	*E*_rev_ (mV)
Controls (*n* = 20)	−1.31 (0.16)	14.8 (0.85)	5.65 (0.71)	81.7 (2.3)
W1538R (*n* = 14)	−1.83 (0.24) *P* = 0.07	14.3 (0.72) *P* = 0.71	6.00 (0.28) *P* = 0.71	82.4 (2.9) *P* = 0.84
A1746G (*n* = 18)	−1.56 (0.27) *P* = 0.42	16.3 (0.64) *P* = 0.17	5.17 (0.36) *P* = 0.58	79.0 (3.2) *P* = 0.51

Data presented as mean values and SEM. *WCC* whole cell capacitance, *E*_*rev*_ Reversal potential. Differences in means of cells with mutant channels compared to wild-type controls were tested by two-tailed Student’s *t* test. *P* < 0.05 was considered significant

We investigated the possibility of ion selectivity being affected by A1746G and W1538R mutations. This was done by calculating the reversal potential (*E*_rev_) for every analyzed cell. On average, *E*_rev_ was not changed by mutation A1746G (79.0 ± 3.2 mV) and W1538R (82.4 ± 2.9 mV) when compared to WT Nav1.7 controls (81.7 ± 2.3 mV). This result suggests that the two investigated mutations have no significant impact on the relative permeability of Na^+^ to Cs^+^ (Table 1).

**Fig. 3:**
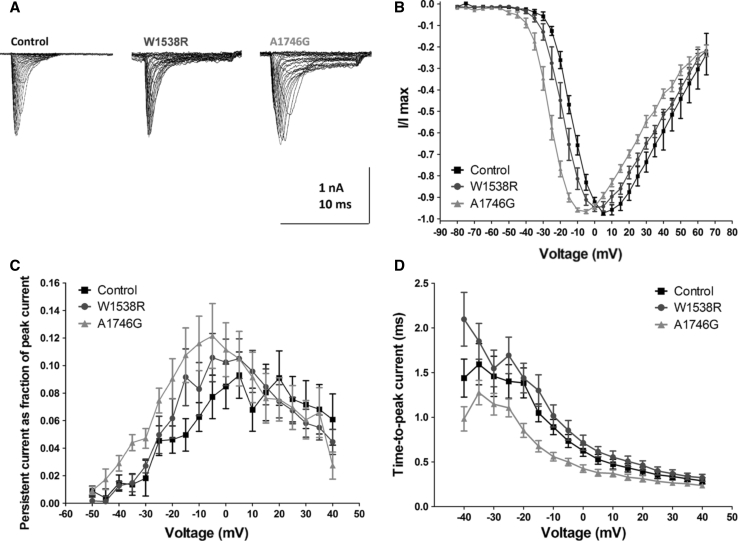
Properties of Nav1.7-mediated currents in voltage-clamp configuration. **a** Example traces from whole-cell voltage-clamp recordings performed in HEK293 cells expressing wild-type Nav1.7 (control, *left*), W1538R mutant (*middle*) or A1746G mutant (*right*) channels. From a holding potential of −120 mV, currents were evoked by voltage increments of 5 mV from −80 to 40 mV. Results from one recording per group are displayed. **b** Normalized current–voltage plots from recordings as described above. **c** Persistent current at 8 ms after onset of the depolarizing voltage step plotted as a fraction of peak currents against voltage. **d** Time-to-peak current as a measure for channel activation kinetics. Data are presented as mean values and SEM (*n* = 20 for controls, *n* = 14 for W1538R and *n* = 18 for A1746G). Wild-type Nav1.7 (Control, *black squares*), W1538R mutation (*blue circles*) and A1746G mutation (*red triangles*)

Voltage dependence of channel activation was found to be changed by both mutations to more hyperpolarizing potentials when plotting peak inward currents (I) as a function of depolarization potential to generate I–V curves (Fig. 3a, b; Table 2).

**Table 2:** Biophysical effects of Nav1.7 mutations W1538R and A1746G

	Steady-state activation		Steady-state fast inactivation		Steady-state slow inactivation		Recovery from steady-state fast inactivation		
	*V*½ (mV)	Slope	*V*½ (mV)	Slope	*V*½ (mV)	Slope	*Tau* (ms)	*K* (1/ms)	*T*½ (ms)
Controls	−8.3 (0.5)	7.3 (0.4)	−51.2 (0.6)	6.9 (0.5)	−7.9 (5.0)	−16.0 (2.6)	11.9 (1.2)	0.08 (0.01)	8.3 (0.9)
W1538R	−16.9 (0.6) *P* < 0.05	6.8 (0.5) ns	−48.9 (0.5) ns	8.1 (0.4) ns	−24.0 (3.6) ns	−13.7 (3.0) ns	10.6 (1.0) ns	0.09 (0.01) ns	7.4 (0.7) ns
A1746G	−23.9 (0.3) *P* < 0.001	6.1 (0.3) ns	−49.8 (0.4) ns	7.1 (0.4) ns	−40.2 (2.9) *P* < 0.01	−13.6 (2.9) ns	7.7 (0.5) *P* < 0.05	0.13 (0.01) ns	5.3 (0.4) ns

Data presented as mean values and SEM. *V* ***½*** Potential of half-maximal activation or inactivation, respectively, *Tau* time constant, *K* rate constant, *T½* Time to half-maximal recovery, *ns* not significant (*P* > 0.05). Differences in means of cells with mutant channels compared to wild-type controls were tested by ANOVA and Bonferroni post hoc test. *P* < 0.05 was considered significant

Quantification of persistent currents as a fraction of peak currents at the time point of 8 ms after the beginning of the depolarizing voltage step revealed a trend to higher values for persistent currents in mutant A1746G, although without statistical significance (Fig. 3c). When investigating time-to-peak as a measure of channel activation rate, we found a trend to shorter time-to-peak as a sign for faster channel activation in A1746G (Fig. 3d). At the voltage step that induced peak current in WT Nav1.7 transfected cells (5 mV), time-to-peak was significantly shorter in A1746G-mutated channels (0.37 ± 0.04 ms) when compared to Nav1.7 WT channels (0.53 ± 0.05 ms; *P* < 0.05).

**Fig. 4:**
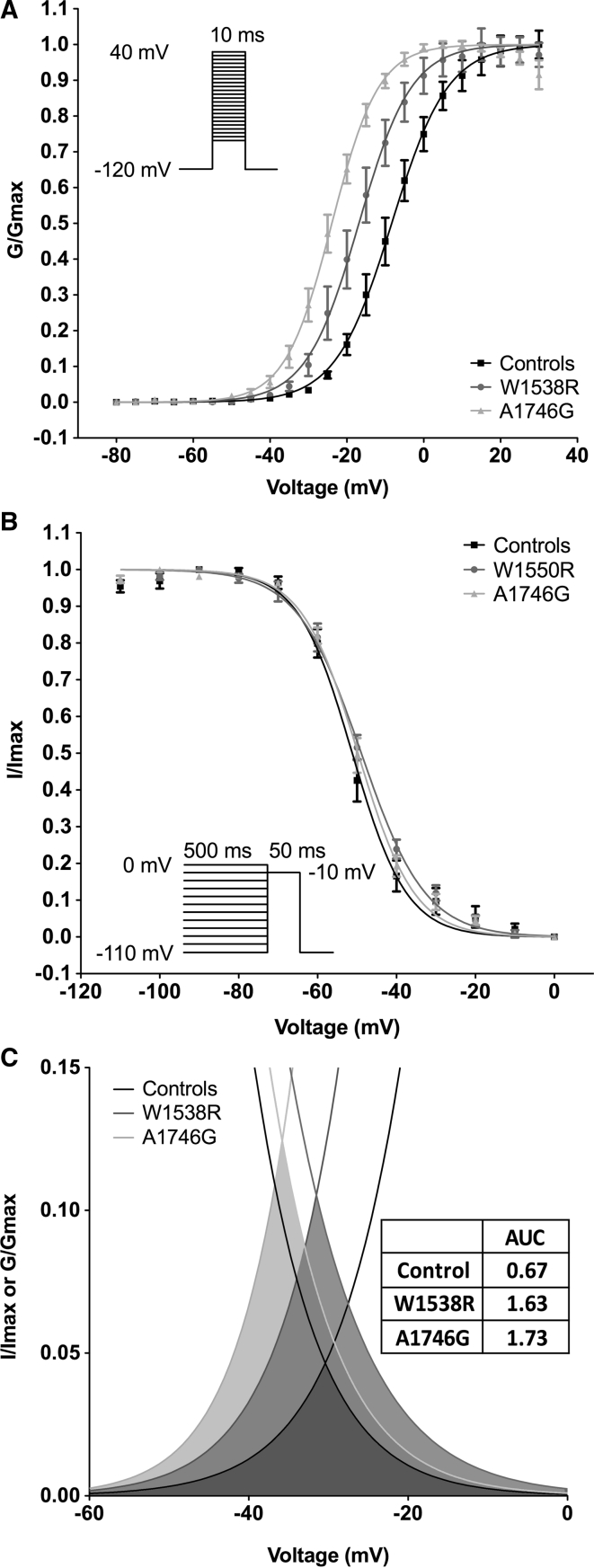
Voltage dependence of steady-state activation and fast inactivation of Nav1.7-mediated currents in voltage-clamp configuration. **a** Conductance values from HEK293 cells expressing wild-type Nav1.7 channels (Control, *black squares*), W1538R mutation (*blue circles*) or A1746G mutation (*red triangles*) were calculated from peak inward currents in activation protocols, normalized and fitted using the Boltzmann equation. Please refer to Table 2 for values for half-maximal activation (*V*½) and slope factors. All data are presented as mean values and SEM (*n* = 20 for controls, *n* = 14 for W1538R and *n* = 18 for A1746G). **b** Voltage dependence of inactivation for wild-type Nav1.7 channels (Control, *black squares*), W1538R mutation (*blue circles*) or A1746G mutation (*red triangles*). Please refer to Table 2 for values for half-maximal inactivation (*V*½) and slope factors. (*n* = 20 for controls, *n* = 13 for W1538R and *n* = 14 for A1746G). **c** Combined analysis of voltage dependence of steady-state activation and fast inactivation in HEK293 cells expressing wild-type Nav1.7 channels (Control, *black lines*), W1538R mutation (*blue lines*) and A1746G mutation (*red lines*). Window currents with increased area under the curve (AUC, see in insert Table) in cells expressing W1538R mutation (*blue area*) or A1756G mutation (*red area*) compared to wild-type controls (*gray area*). Differences in means were tested by ANOVA and Bonferroni post hoc test. *P* < 0.05 was considered significant

Further analysis of steady-state activation revealed a left shift of activation curves in both investigated mutations with significantly different voltages for half-maximal activation (*V*½_act_) for A1746G (−23.9 ± 0.3 mV; *P* < 0.001) and W1538R (−16.9 ± 0.6 mV; *P* < 0.05) when compared to wild-type Nav1.7 channels (−8.3 ± 0.5 mV), while slope factors of voltage-activation relationships were unchanged (Fig. 4a; Table 2).

Steady-state fast inactivation was not found to be affected by A1746G or W1538R mutations, as indicated by largely overlapping curves for voltage dependency of inactivation with similar slope factors and voltages for half-maximal inactivation (*V*½_inact_) not significantly different compared to wild type (Fig. 4b; Table 2).

Combined analysis of the voltage dependence of steady-state activation and fast inactivation showed a marked increase in the size of the window current for both mutations calculated from area under the curves (Controls 0.67; W1538R 1.63; A1746G 1.73) indicative of a gain-of-function mutation (Fig. 4c).

### Mutation of A1746G Leads to Enhanced Slow Inactivation and Faster Recovery from Steady-State Fast Inactivation

**Fig. 5:**
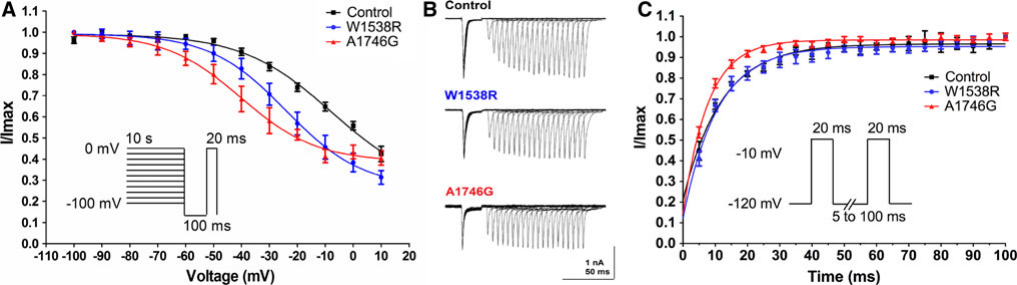
Steady-state slow inactivation and recovery from inactivation of Nav1.7-mediated currents in voltage-clamp configuration. **a** Voltage dependence of steady-state slow inactivation in HEK293 cells expressing wild-type Nav1.7 channels (Control, *black squares*), W1538R mutation (*blue circles*) or A1746G mutation (*red triangles*) was investigated using a 20-ms pulse to 0 mV after a 10-s pre-pulse to potentials from −100 to 0 mV followed by a 100-ms pulse to −120 mV to remove fast inactivation. Resulting currents were then fitted by using the Boltzmann equation. Please refer to Table 2 for values for half-maximal inactivation (*V*½) and slope factors. All data are presented as mean values and SEM (*n* = 16 for controls, *n* = 8 for W1538R and *n* = 7 for A1746G). **b** Representative overlays of example current traces recorded in HEK293 cells expressing wild-type Nav1.7 channels (Controls), W1538R mutation and A1746G mutation. Results from one recording per group are displayed. To investigate the time to recovery from fast inactivation, first, a 20-ms pulse to −10 mV was applied followed by a recovery phase of 5–100 ms and a second pulse to −10 mV. **c** Recorded peak currents were then normalized and fitted using a single exponential equation (one-phase association). Table 2 reports values for time constant (*Tau*), rate constant (K) and half-time (*T*½). All data are presented as mean values and SEM (*n* = 17 for controls, *n* = 10 for W1538R and *n* = 13 for A1746G). Differences in means were tested by ANOVA and Bonferroni post hoc test. *P* < 0.05 was considered significant

Mutation A1746G additionally leads to a hyperpolarizing shift in the observed voltage dependency of steady-state slow inactivation of Nav1.7 (*P* < 0.01) with a *V*½_inact_ of −40.2 ± 2.9 mV compared to −7.9 ± 5.0 mV in WT Nav1.7 (Fig. 5a; Table 2). In contrast, the W1538R mutation of the Nav1.7α subunit did not change this channel property significantly (Fig. 5a; Table 2).

Investigating recovery from steady-state fast inactivation, we found a reduced time constant for recovery of 7.7 ± 0.5 ms for Nav1.7 channels with the A1746G mutation compared to Nav1.7 WT channels with a time constant of 11.9 ± 1.2 ms (*P* < 0.05; Fig. 5b/c). Recovery from steady-state fast inactivation in cells transfected with the W1538R mutation of the Nav1.7 α subunit was not significantly different from WT channels (Fig. 5b/c; Table 2).

In silico simulation of the observed biophysical changes caused by mutation A1756G and W1538G indicated a reduction in current injection thresholds for induction of a single action potential (Supplementary material 2). The threshold for induction of action potential firing after simulated current injection of 225 pA in wild-type control was reduced to 115 pA (−49 %) by simulating mutation A1746G, while mutation W1538R had a threshold of 144 pA (−36 %). We found an increase in action potential firing frequency (Supplementary material 2). At a simulated current injection of 250 pA, mutation A1746G increased the number of action potentials within 200 ms from 1 to 20, while mutation W1538R resulted in 13 action potentials (Supplementary material 2).

In summary, simulation of mutation A1746G was associated with greater changes in both investigated parameters compared to wild-type Nav1.7, while mutation W1538R induced less pronounced differences in action potential firing frequency and current threshold. Both simulated mutations lead to increased neuronal excitability indicative of a gain-of-function mutation.

## Discussion

So far, all primary erythromelalgia (PE) SCN9A-related mutations that were characterized electrophysiologically have been located in the first three domains of Nav1.7 with the main cluster of mutations in the S4-S5 region of the second domain (DII) (Choi et al. 2006; Lampert et al. 2010). We report the effect of two mutations within the fourth domain of Nav1.7 on sodium channel function (W1538R in S2 of DIV and A1746G in S6 of DIV). The half-point of activation was shifted toward more hyperpolarized potentials, while steady-state fast inactivation was not different in both mutants. The hyperpolarizing shift in steady-state activation results in a greater overlap of steady-state activation and fast inactivation, which is often referred to as window currents (Wedekind et al. 2001). We have observed an enhanced slow steady-state inactivation and a shorter recovery from steady-state inactivation in channels with the A1746G mutant only, while these properties were normal in channels with W1538R mutation. These findings are overall consistent with those observed in other SCN9A mutations causative for primary erythromelalgia (Dib-Hajj et al. 2010). Finally, in silico simulations of both mutations in a small-diameter neuron revealed reduced thresholds for action potential firing and enhanced repetitive firing, indicating hyperexcitability caused by biophysical channel property changes by mutation W1538R and A1746G. Since the patient with the A1746G mutation showed a positive response to systemic treatment with the sodium channel blocker mexiletine, it might be useful to elucidate the effects of mexiletine on Nav1.7 channels with this mutation in vitro.

### Spectrum of Inherited Gain-of-Function Conditions Mapped to SCN9A

The original range of inherited conditions associated with SCN9A (i.e. PE, PEPD and CIP) has recently been supplemented with chronic non-paroxysmal neuropathic pain (Dabby et al. 2011), small fiber neuropathies (SFN) (Faber et al. 2012) and partial congenital insensitivity to pain (Staud et al. 2011; Yuan et al. 2011). It is as yet not fully understood why some gain-of-function mutations in SCN9A are found to cause small fiber neuropathy, while others cause gain-of-function but not small fiber neuropathy (Faber et al. 2012; Han et al. 2012), as in the patient reported here with the W1538R mutation, in whom intra-epidermal nerve fiber density was found to be normal.

### Different Phenotypes Attributed to the Same Mutation

Dabby and co-authors have attributed chronic non-paroxysmal neuropathic pain to the W1538R mutation (Dabby et al. 2011), while our patient 1 carrying the same mutation had exhibited classic clinical features of primary erythromelalgia. In a similar fashion, Estacion and colleagues have reported three different clinical presentations in patients genotyped for the same I228 M substitution in Nav1.7 (Estacion et al. 2011). One patient presented with severe facial pain, while the other sufferer from the same family had distal pain in hands and feet. The third and unrelated subject was complaining of discomfort in the scalp region.

Complex phenotypes representing a combination of pathological conditions and the same mutations manifesting differently in the same and different families are not exclusive to SCN9A (Estacion et al. 2011). For example, mutations in the predominantly cardiac isoform of VGSC family, SCN5A, can be represented by phenotypes combining long QT and Brugada syndromes (Bezzina et al. 1999), or a combination of long QT syndrome and congenital heart block (Lupoglazoff et al. 2001).

In a wider medical context, when a disease-causing mutation is expressed, it is not necessarily expressed in all affected individuals in the same way. These variable phenotypes, such as the one described here, may be caused by a number of factors, such as gene–environment interactions, gene modifiers (genetic background possibly affecting penetrance, dominance or expressivity) and allelic variation among others (Janku et al. 1980; Collins et al. 1993; Muenke et al. 1994; Nadeau 2001). Whether the observed phenotypic diversity between the two carriers of the same mutation, W1538R, is attributed to epigenetic, environmental factors or gene modifiers remains unknown (Estacion et al. 2011). However, the common feature of this mutation appears to be the relatively late onset of clinical manifestation.

### Coupling of Activation–Inactivation

Each domain of the α unit of VGSC consists of 6 transmembrane segments (S1-S6). The first four, S1 to S4, are believed to form the voltage sensing part of the channel, while the channel pore comprises S5-S6. When aligning the sequences of the four domains contributing to the α subunit, the sequence of DI is similar to DIII, while DII is similar to DIV. It is therefore thought that sodium channels developed evolutionary from a one domain precursor via a two-domain structures as an intermediate developmental step (Charalambous and Wallace 2011).

The only previously described lesion mapped to DIV is mutation A1632E, which in fact has features overlapping two inherited conditions—PEM and PEPD. It is mapped to S4-S5 intracellular linker of DIV (Estacion et al. 2008). Both patients described here had clinical symptoms strongly consistent with those of primary erythromelalgia, but no features suggestive of PEPD. It has been proposed that based on our current understanding derived from mapping gain-of-function mutations in the SCN9A gene, the channel activation process is associated with DI and DII (Estacion et al. 2008), while inactivation or coupling of activation–inactivation is linked to DIII and DIV conformational changes (Ma et al. 2009). Intriguingly, a recent publication by Waxman and co-workers demonstrated that two different mutations, located within the DIII/S4-S5 linker region and only 10 amino acids apart from each other, lead to distinct biophysical effects (enhancement of activation or inactivation, respectively) and therefore distinct pain disorders (PEM and PEPD). This once more indicates that the effect of a particular mutation on channel function may be more relevant than its location within the channel protein (Cheng et al. 2010). So far, W1538R and A1746G are the only mutations mapped to DIV that have been found to predominantly affect the channel property of steady-state activation, while steady-state fast inactivation is unchanged. The W1538 residue is conserved only in three of the nine known human voltage-gated sodium channels. Interestingly, an arginine (R) at the corresponding position in Nav1.1 and Nav1.3 is documented as the normal variant (Fig. 2).

### Correlation Between Age of Onset and Phenotype with Differences Found in Biophysical Properties of Nav1.7 Mutations

A correlation between the age of onset and the magnitude of the hyperpolarizing shift in voltage dependence of steady-state activation of Nav1.7 has been proposed previously (Cheng et al. 2008; Han et al. 2009), although alternative splicing has been proposed to have an impact on age of PEM manifestation as well (Choi et al. 2010). In recent reviews, it has been suggested an association between the degree of steady-state activation alteration in particular with early age of onset (0–16 years) of clinical symptoms typical for primary erythromelalgia (Han et al. 2009; Lampert et al. 2010). The patient we describe here with the W1538R mutation had an extremely late onset of symptoms after the age of 61. Prior to molecular genetic testing for SCN9A mutations and given the lack of a clear family history, she would have previously been diagnosed as having secondary erythromelalgia. We have not seen any other cases with such late onset reported in the literature. Few detailed QST profiles have been reported in patients with primary erythromelalgia and confirmed SCN9A mutations. She demonstrated a mix of reduced sensitivity to thermal stimuli as well as a gain-of-function in relation to mechanical hyperalgesia and dynamic mechanical allodynia. There was no loss of intra-epidermal nerve fibers to explain the raised cooling detection thresholds and reduced thermal sensory limen. Microneurographic recordings from patients suffering from erythromelalgia demonstrate ongoing activity in primary afferent nociceptors (Orstavik and Jørum 2010). The mechanical hypersensitivity which we observed may be due to altered transduction properties of primary afferents to mechanical stimuli or central sensitization as a consequence of ectopic activity. This QST profile is quite different to a patient we recently described with an L858F mutation (Segerdahl et al. 2012), who demonstrated selective thermal hyperalgesia and further studies of such patients are needed to see whether particular QST profiles may relate to certain SCN9A mutations or whether the QST profile may change over time.

## Conclusions

In conclusion, we clinically and biophysically characterized two novel mutations within DIV of Nav1.7 encoded by SCN9A, both leading to typical phenotypes of primary erythromelalgia. While we found that mutation A1746G led to marked changes in biophysical properties of Nav1.7 and early onset of clinical symptoms, mutation W1538R resulted in less pronounced consequences on evaluated channel properties and a very late onset of disease. This finding demonstrates that mutations encoding for DIV of Nav1.7 can not only be linked to CIP or PEDP but also be causative of primary erythromelalgia.

## Electronic supplementary material

Below is the link to the electronic supplementary material.

Supplementary material 1 (DOC 469 kb)

## Abbreviations

A/D: Analog to digital
CIP: Congenital insensitivity to pain
CT: Computer tomography
D: Domain
dbSNP: Single nucleotide polymorphism database
DFNS: German Research Network on Neuropathic Pain
DMEM: Dulbecco’s modified Eagle’s medium
DN4: Douleur Neuropathique en 4 Questions
DNA: Deoxyribonucleic acid
DRG: Dorsal root ganglion
EGTA: Ethylene glycol tetraacetic acid
ECG: Electrocardiography
EEG: Electroencephalography
EMG: Electromyography
*E*_rev_: Reversal potential
GFP: Green fluorescent protein
HEK 293: Human embryonic kidney cells
HEPES: 4-(2-Hydroxyethyl)-1-piperazineethanesulfonic acid
I: Current
IEM: Inherited erythromelalgia
IRES: Internal ribosome entry site
Nav: Voltage-gated sodium channel
PEM: Primary erythromelalgia
PEPD: Paroxysmal extreme pain disorder
QST: Quantitative sensory testing
S: Segment
SEM: Standard error of mean
*V*: Voltage
WT: Wild-type
