# Supplementary material for: Novel Mutations Mapping to the Fourth Sodium Channel Domain of Nav1.7 Result in Variable Clinical Manifestations of Primary Erythromelalgia
Source: Neuromolecular Med. 2013 Jan 6;15(2):265–78. doi: 10.1007/s12017-012-8216-8 (PMC3650253; doi:10.1007/s12017-012-8216-8)
Supplement: Supplementary file 1 — Supplementary material 1 (DOC 469 kb) [file 12017_2012_8216_MOESM1_ESM.doc]

**Supplementary Material**

**Supplementary Material 1 – Thermographic data of patient 2**


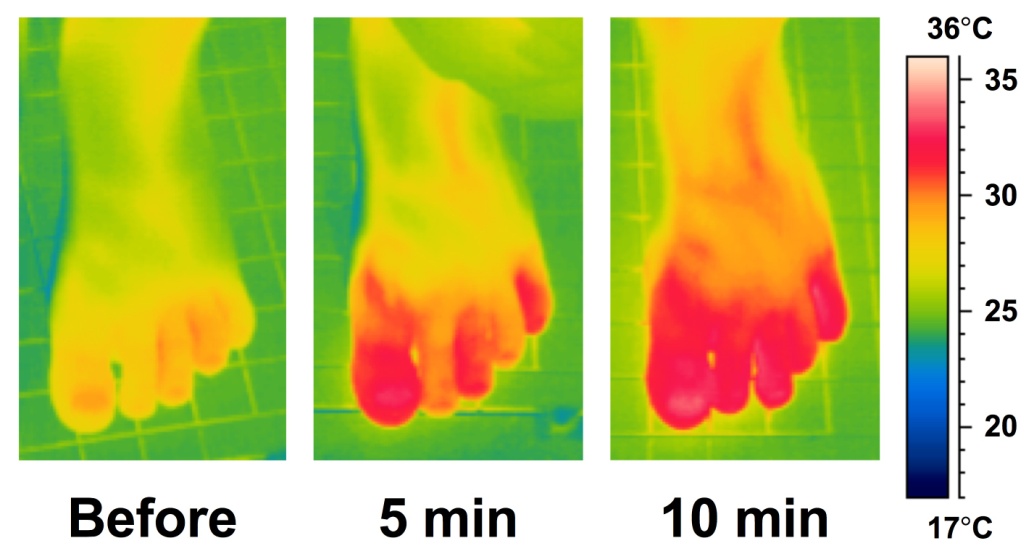


Supplementary Figure 1. Thermography was performed on patient 2 using a FLIR A320G thermal imager (FLIR Systems, West Malling, UK), with ethernet connection to a PC running FLIR Thermacam Researcher software for thermal image capture. The patient removed shoes and socks and sat for 15 minutes prior to imaging at a room temperature of 23 degrees Celsius. A baseline thermogram of the feet was then recorded (Before). The feet were then placed in thin plastic bags and immersed for one minute in water at 15 degrees Celsius. After the cold challenge, the feet were removed from the water and bags, and further thermographic images were recorded after 5 and 10 minutes to assess the rate of foot rewarming. The results revealed a rapid hyperaemic response to cold challenge.

Supplementary Material 2 - Simulation of a DRG neuron displaying the electrophysiological properties of wildtype and mutant Nav1.7

The NEURON programming environment (version 7.2; Neuron Project, Yale University), a software for empirically-based simulations of neurons and networks of neurons was used for simulating electronic properties of a sensory neuron (Hines & Carnevale, 1997) as previously described (Sheet*s et a*l., 2007). A mathematical model of an isolated neuron described previously (Spampanat*o et a*l., 2004; Barel*a et a*l., 2006) was compiled and adjusted to simulate a small-diameter neuron using a single compartment cylindrical model with a diameter of 20 μm and a length of 20 μm. Sodium and delayed rectifier potassium channels were included as described previously (Spampanat*o et a*l., 2004). The models included either wild-type Nav1.7, W1538R mutant channels or A1746G mutant channels with voltage dependencies of activation, fast inactivation and slow inactivation as characterized in this study. Voltage-dependency of steady-state sodium channel activation was modelled using the function m_∞_(V)^Nav1.7^ = 1/{1+exp[-(V-V_½act_)/Slope]} with V_½act_ being the potential leading to half-maximal current activation in whole-cell recordings. Voltage-dependent fast and slow inactivation was described by h_∞_(V)^Nav1.7^ = 1/{1+exp[(V-V½f_inact)/Slope]} and s_∞_(V)^Nav1.7^ = 1/{1+exp[(V-V½s_inact)/Slope]} with V½f_inact and V½s_inact being defined as the potentials leading to half-maximal current inactivation in whole-cell recording protocols for evaluation of steady-state fast and slow inactivation, respectively. Kinetics of activation and fast inactivation were used to simulate time-dependency of conductance as described before (Spampanat*o et a*l., 2004). Simulation of slow inactivation kinetics was based on wild-type values reported previously (Vijayaragava*n et a*l., 2001). The description of kinetics used for all sodium channels represent those that were determined with co-expression of the Navβ1 and Navβ2 subunit. The kinetics of the delayed rectifier potassium channels were the same as that used previously (Spampanat*o et a*l., 2004). Initial resting membrane potential was set to -60 mV and stimulation was started with a delay of 50 ms. Reversal potential for potassium and sodium for all conducted simulations were set to -70 mV and +65 mV, respectively.

To investigate the effect of the mutations on the current threshold for inducing a single action potential, currents were injected in increments of 1 pA. We found a reduction of current injection thresholds for induction of a single action potential (Supplementary Figure 2), both indicative of an increased excitability of the simulated neuron. The threshold for induction of action potential firing after simulated current injection of 225 pA in wildtype control was reduced to 115 pA (-49%) by simulating mutation A1746G, while mutation W1538R had a threshold of 144 pA (-36%).

To evaluate the consequences of the mutations for action potential firing frequency, a current injection between 0 and 500 pA in increments of 50 pA for a duration of 200 ms was simulated and the number of resulting AP analysed. We found an increase of action potential firing frequency (Supplementary Figure 3). At a simulated current injection of 250 pA, mutation A1746G increased the number of action potentials within 200 ms from 1 to 20, while mutation W1538R resulted in 13 action potentials (Supplementary Figure 3A).

In summary, simulation of mutation A1746G was associated with greater changes in both investigated parameters compared to wildtype Nav1.7, while mutation W1538R induced less pronounced differences in action potential firing frequency and current threshold. Both simulated mutations lead to increased neuronal excitability indicative of a gain-of-function mutation.

**Supplementary Figure 2**


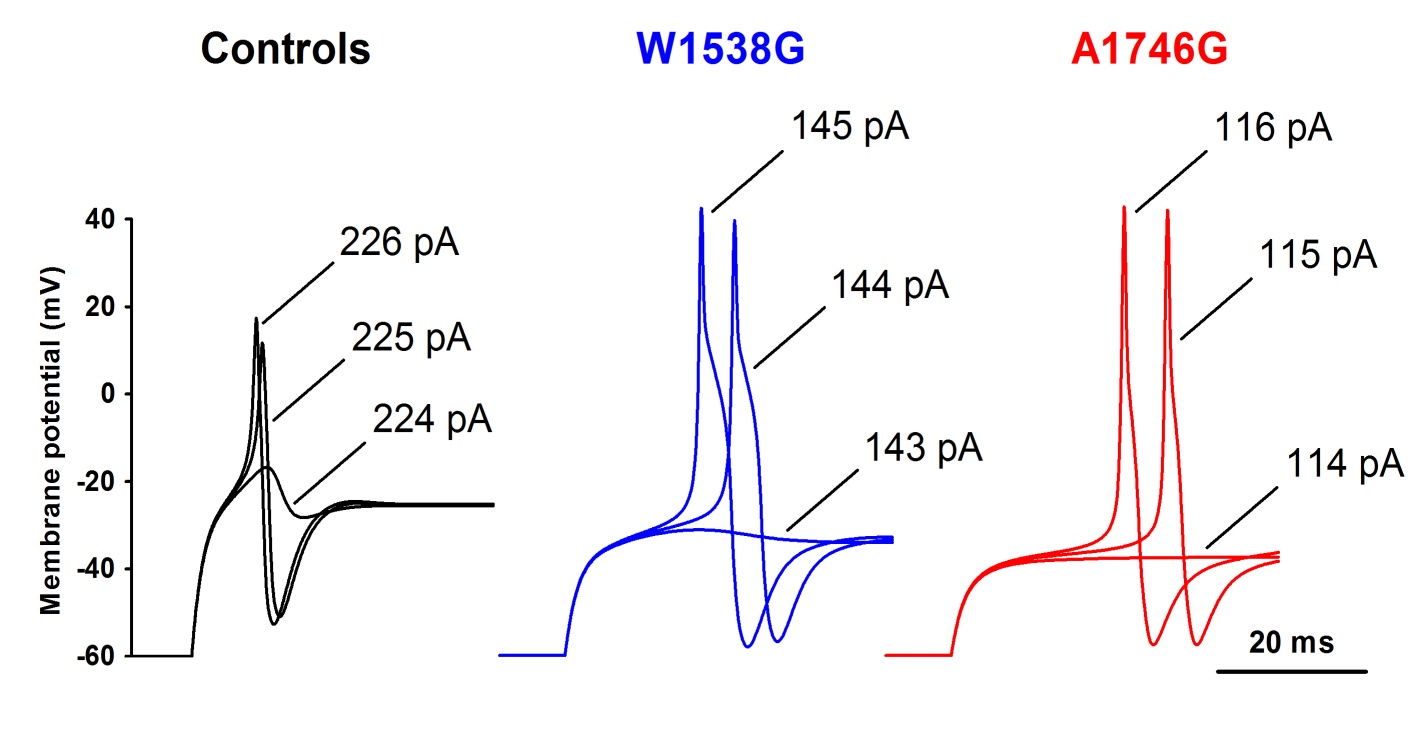


Supplementary Figure 2. Current thresholds inducing single action potential firing in DRG neuron simulation.

Resulting traces from in silico small-diameter neuron simulation of wildtype Nav1.7 (controls), W1538R and A1746G mutations are depicted. After determining current injection thresholds for induction of a single action potential using increasing current injection with increments of 1 pA, 3 traces were recorded separately and merged to an overlay for this illustration – one sub-threshold, and two subsequent stimulations leading to action potential firing. This overlay is not illustrating firing frequency as these are single, superimposed action potentials. Lower current thresholds were observed with both mutations.

**Supplementary Figure 3**


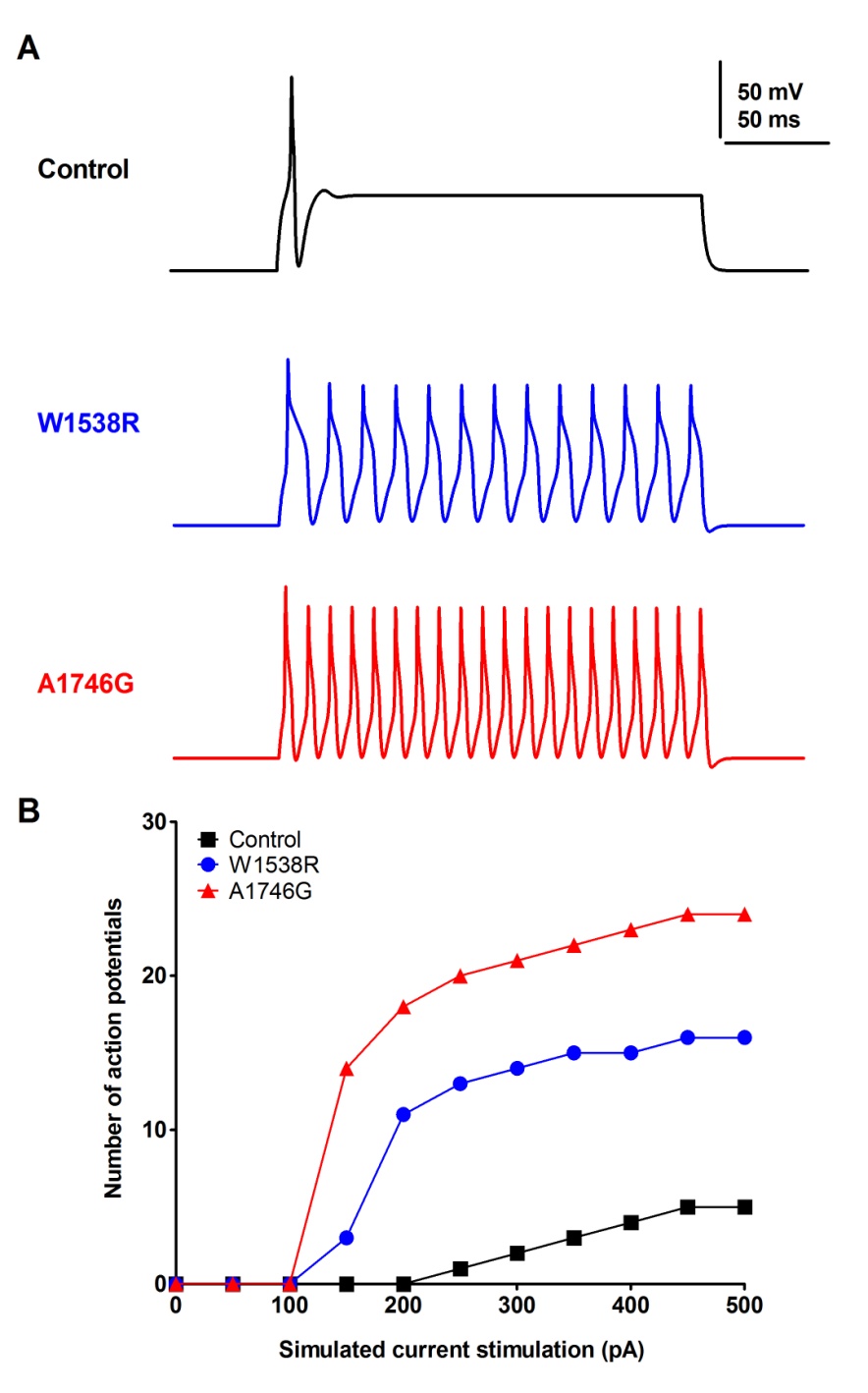


Supplementary Figure 3. Action potential firing frequency in DRG neuron simulation.

(A) Action potential firing pattern in a small-diameter neuron model simulation with wildtype Nav1.7, W1538R or A1746G mutation. A simulated current of 250 pA was injected for 200 ms and the number of predicted action potentials counted. (B) Representation of the number of action potentials generated within 200 ms in response to injection of different currents in the in silico neuron. Current injections ranged from 0 to 500 pA with increments of 50 pA.

**Supplementary Reference List**

Barela, A.J., Waddy, S.P., Lickfett, J.G., Hunter, J., Anido, A., Helmers, S.L., Goldin, A.L., & Escayg, A. (2006) An epilepsy mutation in the sodium channel SCN1A that decreases channel excitability. *J. Neurosci.*, **26**, 2714–2723.

Hines, M.L. & Carnevale, N.T. (1997) The NEURON simulation environment. *Neural Comput*, **9**, 1179–1209.

Sheets, P.L., Jackson, J.O., 2nd, Waxman, S.G., Dib-Hajj, S.D., & Cummins, T.R. (2007) A Nav1.7 channel mutation associated with hereditary erythromelalgia contributes to neuronal hyperexcitability and displays reduced lidocaine sensitivity. *J. Physiol. (Lond.)*, **581**, 1019–1031.

Spampanato, J., Aradi, I., Soltesz, I., & Goldin, A.L. (2004) Increased neuronal firing in computer simulations of sodium channel mutations that cause generalized epilepsy with febrile seizures plus. *J. Neurophysiol.*, **91**, 2040–2050.

Vijayaragavan, K., O’Leary, M.E., & Chahine, M. (2001) Gating properties of Na(v)1.7 and Na(v)1.8 peripheral nerve sodium channels. *J. Neurosci.*, **21**, 7909–7918.
